# Independent and synergistic effects of microplastics and endocrine‐disrupting chemicals on the reproductive social behavior of fathead minnows (*Pimephales promelas*)

**DOI:** 10.1002/ece3.10846

**Published:** 2024-02-06

**Authors:** Grace Carter, Jessica Ward

**Affiliations:** ^1^ Biology Department Ball State University Muncie Indiana USA

**Keywords:** EE2, microplastics, reproductive behavior, vector hypothesis

## Abstract

Microplastics (MPs) have become an environmental concern in recent years, with most research focused on the physiological effects of exposure. Comparatively little consideration has been given to the potential behavioral impacts of exposure, which may also have fitness consequences for individuals. Moreover, MPs can serve as vectors for endocrine‐disrupting chemicals and other locally co‐occurring contaminants known to impair behavioral responses. This project aimed to determine whether MPs alone or in association with a common environmental EDC (17‐alpha ethinyl estradiol; EE2) alter reproductive behavior and decision‐making in fish. Male and female fathead minnows (*Pimephales promelas*) were exposed to MPs associated with either a low (10 ng/L; MP_EE2 10_) or high (50 ng/L, MP_EE2 50_) concentration of EE2, or MPs without EE2 (MP_virgin_) for 30 days via a dietary feeding protocol. Behavioral trials were conducted on Day 31 to determine the effects of exposure on male–female social interactions. The expression of male sexually selected traits, including courtship, was unaffected by exposure. However, non‐exposed females in all treatment groups trended toward discrimination against exposed males, which reached statistical significance for the MP_EE2 50_ group. Female fish exposed to MPs, alone or in association with EE2, were equally likely to approach and associate with non‐exposed and exposed males. The results from this study suggest that MPs may alter social behavior in fishes and that the behavioral impacts of exposure may be more strongly pronounced in females than males. Such individual‐level changes in fitness have the potential to impact population size, with downstream effects on the broader aquatic community.

## Graphical abstract

Microplastics (MPs) and endocrine‐disrupting chemicals can alter the behavior of aquatic organisms, but this is little studied. This study evaluated the effects of MPs and estrogen on social interactions between male and female fathead minnows. Exposure to MPs, alone or in combination with EE2, had few observable effects on male behavior but non‐exposed female fish discriminated against exposed males.

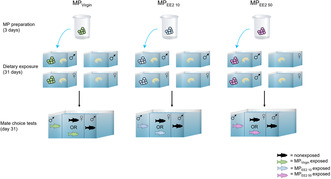

## 1 INTRODUCTION

Microplastics (MPs) are defined as plastic particles of <5 mm in diameter that are insoluble in water and enter the environment via the deterioration of discarded synthetic clothes, containers, tires, and single‐use plastics, or as a component of industrial and commercial cleaning products (Baldwin et al., 2016; Browne et al., 2007; Foley et al., 2018; Frias & Nash, 2019). MPs are ubiquitous in both marine and freshwater environments (Eerkes‐Medtrano et al., 2015), and are found globally in benthic substrates, within the water column, and at the surface of water (Stanton et al., 2020; Wright et al., 2013; Zhao et al., 2015). The small size of MPs allows for easy migration of particles into aquatic organisms at lower trophic levels via unintentional ingestion or respiration (Barboza, Cózar, et al., 2019; Crawford & Quinn, 2017). The subsequent consumption of fishes and other affected organisms can lead to the bioaccumulation of MPs in higher trophic levels (Batel et al., 2016; Brennecke et al., 2015) and present a potential health risk to humans (Barboza, Lopes, et al., 2019).

In addition to their potential effects on humans, exposure to MPs also has negative impacts on the growth, physiology, and reproduction of affected aquatic organisms (Baldwin et al., 2016; Foley et al., 2018). For example, ingestion of MPs reduces growth rate and decreases fecundity in freshwater amphipods (Au et al., 2015). In *Daphnia* spp., exposure to MPs causes immobilization (Rehse et al., 2016). Adverse effects have also been observed in fishes and other aquatic vertebrates, ranging from digestive system blocks to decreases in growth and declines in reproduction (Jabeen et al., 2018; Qiang & Cheng, 2020; Wright et al., 2013). However, despite growing knowledge and concern about the distribution, fate, and biological effects of MPs on fish, substantial research gaps remain. Notably, most current research on the biological impacts of MPs has focused on physiological effects on animals. However, sublethal behavioral changes in the performance of individuals due to contaminants (Clotfelter et al., 2004; van der Sluijs et al., 2011) or other forms of anthropogenic disturbance (Tuomainen & Candolin, 2011) are well documented. Such changes in behavior following exposure to contaminants can translate into population and community declines (Kidd et al., 2007, 2014; Rearick et al., 2018). In addition, little is known about the independent and synergistic impacts of MPs and other environmental stressors on individuals, populations, and communities (Swank et al., 2022). For example, growing evidence suggests that the hydrophobic nature and large surface area of MP particles allows for organic contaminants, heavy metals, endocrine‐disrupting chemicals (EDCs), and microbiota to sorb to the surface of the particles (Caruso, 2019; Wang, Ge, & Yu, 2019), which then act as an additional vector of exposure for these compounds (Wang, Ge, & Yu, 2019; Wu et al., 2016). The plastics themselves may also contain additives that leach into the body, with adverse effects on aquatic organisms (Liu et al., 2019).

One group of compounds known to impair behavior are EDCs. EDCs act by increasing or blocking hormones through hormone receptors (Tabb & Blumberg, 2006). One way that water bodies may be contaminated by EDCs is through deposition of human metabolic waste. Wastewater treatment plants may not fully remove these contaminants from effluent, which are then dispersed in the environment (Vilela et al., 2018). The negative effects of EDCs on the physiology and behavior of aquatic organisms are well known (Arukwe, 2001; Cram et al., 2019; Dzieweczynski et al., 2018; Saaristo et al., 2019). For example, exposure to environmental estrogens decreases male courtship behavior in fish species (Lavelle & Sorensen, 2011; Salierno & Kane, 2009) and alters the outcome of intraspecific reproductive social interactions, with non‐exposed female fish frequently being more responsive to non‐exposed males compared to EDC‐exposed males (Baatrup & Henriksen, 2015). Such changes in reproductive behavior and decision‐making are important because they have the potential to drive changes in the phenotypic composition and evolutionary trajectory of a population (Söffker & Tyler, 2012).

This research aimed to determine whether (i) exposure to MPs alters male–female social behavior during reproductive interactions in the fathead minnow (*Pimephales promelas*) and (ii) MPs act as a vector for other contaminants known to affect reproductive behavior. *Pimephales promelas* are a common Cyprinid found in lotic and lentic environments across North America. We selected this species as our model organism because their reproductive behavior is well understood and the species is commonly used in ecotoxicology studies, making it feasible to compare effects of exposure across EDCs and fitness contexts. Relevant to this study, individuals rapidly reach sexual maturity and reproductive motivation is easily induced in the laboratory (Ankley et al., 2001).

In nature, *P. promelas* males defend nests composed of a floating or submerged substrate (Unger, 1983). Males are typically larger than females, with males weighing 3–5 g, compared to 2–3 g for females (Ankley & Villeneuve, 2006). During the breeding season, males compete for, maintain, and defend appropriate mating substrates. Courtship behaviors include rapidly approaching the female, stopping in front of the female, and leading the female to a breeding tile. Males will also attempt to make contact with females by nudging the female in the side (Cole & Smith, 1987). Sexually mature, reproductively motivated male minnows also display several secondary sexual traits, including melanic body coloration consisting of three vertical, dark bands on the lateral sides of body, and the presence of tubercles on the anterior head and snout (McMillan & Smith, 1974). The development and intensity of expression of these traits are regulated by testosterone, and trait expression is commonly decreased following exposure to estrogenic EDCs (Ankley et al., 2001).

We selected 17‐alpha ethinyl estradiol (EE2; a common environmental estrogen; Saaristo et al., 2019) as our model contaminant for this study because it has well‐established effects on male and female behavior, and on the expression of male secondary sexual traits known to affect female choice decisions. Previous research has shown that female exposure to EE2 may increase receptivity in females when choosing a mate (Cram et al., 2019; Saaristo et al., 2019). However, non‐exposed females discriminate against males that have been exposed to environmental estrogens based on changes in male courtship (Dzieweczynski & Kane, 2017). Here, we exposed both male and female minnows to MPs for 30 days, and then tested them in dual‐choice trials to determine whether exposure to MPs, alone or in conjunction with EE2, impaired male courtship behavior and/or the outcome of female mate choice decisions. To our knowledge, this study is the first to assess how MPs, alone or as a vector for EDCs, alter complex male–female reproductive social interactions.

## 2 METHODS

### 2.1 Animals, maintenance, and housing

This study was conducted in a dedicated aquatic laboratory facility under controlled conditions at Ball State University between November 2021 and December 2023. Adult *P. promelas* (8–9 months) were shipped to the laboratory from an aquatic laboratory facility (Aquatic Biosystems Inc.) at regular intervals (~3 months). A total of 288 fish were used in this experiment, with 48 individuals used for each treatment. Prior to experiments, fish were separated by sex and housed in a 605 L living stream divided by mesh barriers. The stock tank was outfitted with filters and air stones to ensure proper aeration and oxygenation. The fish were fed *Artemia* spp. and bloodworms twice a day ad libitum. The fish were kept under summer breeding conditions in all stock and experimental tanks during the experiment (21–23°C and a 16:8 h light:dark photoperiod). Temperature and total dissolved solids were monitored daily to ensure that healthy conditions were maintained in the housing tanks (Ankley & Villeneuve, 2006).

### 2.2 MP treatments and dietary exposure

Methods for the preparation of exposure treatments and exposure protocol followed Swank et al. (2022). This project used clear polyethylene microspheres (300–350 μm, Cospheric). Polyethylene was selected for this experiment because it is common in aquatic environments (Browne et al., 2011). Before the start of the experiment, microspheres were placed in a 75‐μm nylon micromesh bag and submerged in water containing an environmentally relevant low (10 ng/L) or high (50 ng/L) concentration of EE2, or clean water absent of EE2, hereafter referred to as virgin MPs. Concentrations of EE2 in the aqueous solution were determined via LC–MS/MS (SGS AXYS Analytical Services). Microspheres were soaked for 72 h at room temperature to ensure maximal sorption of estrogen (Wu et al., 2016), and the water was exchanged every 24 h to minimize EE2 degradation. After 72 h, the MPs were removed from solution, air‐dried, weighed into feeding aliquots (10.4 mg or ~500 MPs), and frozen at −20°C until use in the experiment.

**FIGURE 1:**
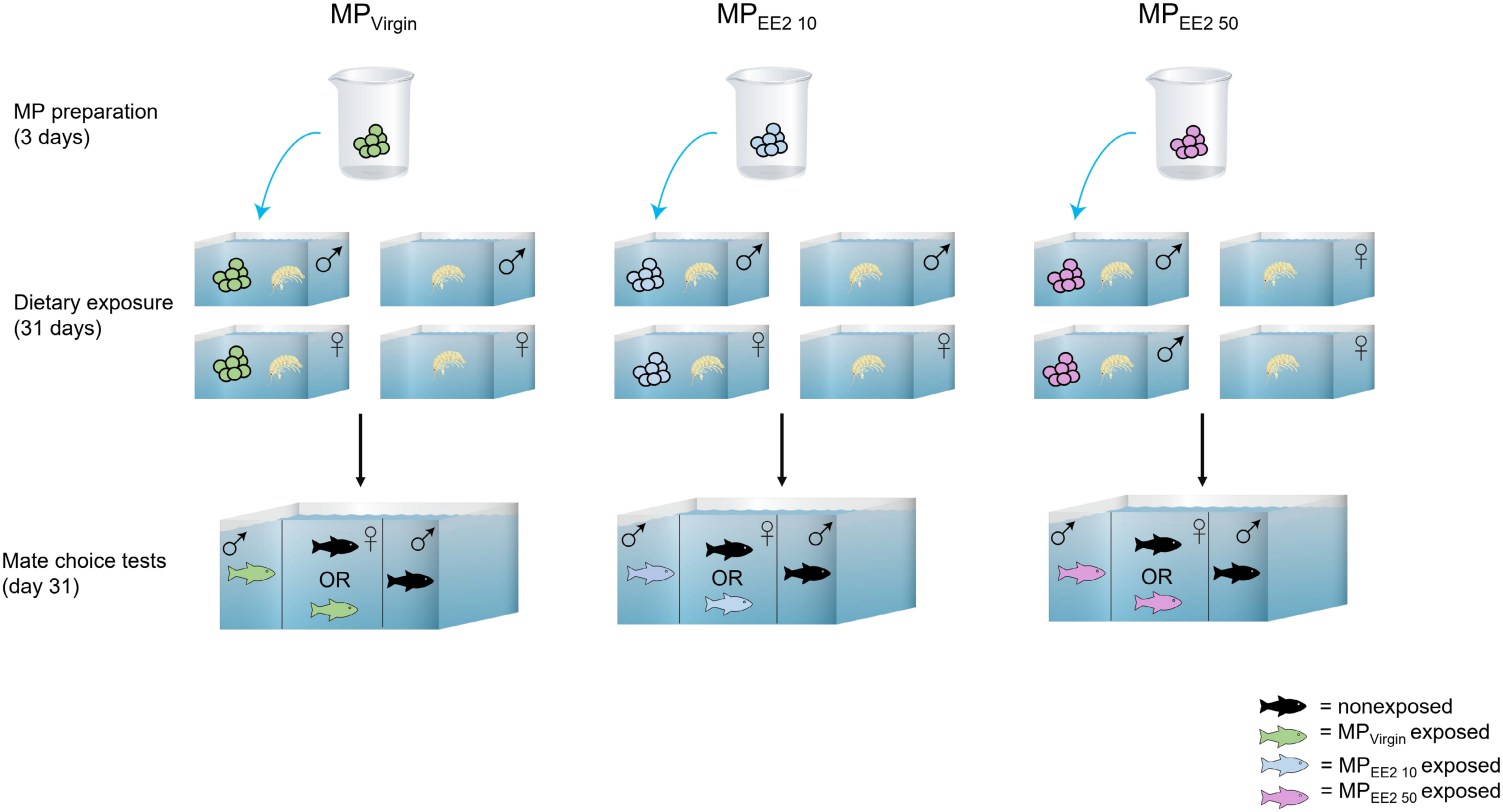
Experimental design. Microplastics (MPs) were prepared before the experiment by immersing the particles in either a low or a high concentration of 17‐alpha ethinyl estradiol (EE2; MP_EE2 10_ or MP_EE2 50_), or water (MP_Virgin_), for 72 h. Separately housed groups of males and females underwent a twice‐daily dietary exposure regime for 30 days, consisting of live brine shrimp mixed with MPs (100 MPs/L). Control fish received only brine shrimp. Reproductive social interactions between males and females were assessed on Day 31 of the experiment. Black fish represent non‐exposed (control) fish. Green, blue, and purple fish represent those exposed to MP_Virgin_, MP_EE2 10_, and MP_EE2 50_, respectively.

Six replicate exposures were conducted, with each exposure replicate culminating in a maximum of four behavioral choice trials for each treatment. For each replicate, the exposure start dates for each treatment were staggered by 1 day to allow all behavioral tests to be conducted on Day 31 of exposure. At the start of each replicate, four males and four females were selected from the stock tank and assigned to one of the exposure treatments: MP_Virgin_, MP_EE2 10_, or MP_EE2 50_ (eight fish in total exposed per treatment per replicate). Another eight fish (four males and four females) were selected from the stock tank to serve as the corresponding controls. Therefore, each exposure replicate involved 48 total fish (8 fish per treatment × 3 treatments × 2 (control and exposed groups) = 48 fish/replicate; Figure 1). Each group of four individuals was maintained in a 38‐L housing tank (51 cm × 27 cm × 32 cm). Each tank contained two PVC shelters and was separated by treatment group and sex. Tanks were arranged such that males and females did not have visual access to any individual with whom they would be tested in behavioral trials. However, males were permitted visual access to control females from other treatment sets to maintain reproductive motivation throughout the exposure period.

The fish underwent twice‐daily exposures following an established dietary feeding protocol (Swank et al., 2022). Exposures were separated by at least 7 h to ensure motivation to forage. At the start of an exposure event, the four fish housed together were introduced into a feeding tank containing 5 L of aged water. Control fish were given approximately 1 mL of live *Artemia* spp. Exposed fish were given an equivalent amount of brine shrimp mixed with virgin MPs (MP_Virgin_; exposure concentration 100 MPs/L) or particles associated with either the low or high concentration of EE2 (MP_EE2 10_ and MP_EE2 50_). All fish were permitted to forage naturally for 30 min before being moved back to their home tanks. An air stone in the tank ensured that MPs remained in suspension for the entirety of exposure period. At the end of each dietary exposure event, the feeding tanks were drained and any MPs remaining in the tank were collected. The tanks were filled with fresh aerated water before the next feeding. Dietary exposures were conducted for 30 consecutive days, and fish were tested in behavioral trials on Day 31 of the experiment.

### 2.3 Behavioral trial procedures

Behavioral tests followed methods outlined in TerMarsch and Ward (2020). Tests were conducted in four 75.7‐L dual‐choice trial tanks (77.5 cm × 33 cm × 33 cm). These trial tanks were lined with gravel and divided into three compartments separated by opaque and mesh dividers. Photographs of natural vegetation covered the back and side walls. Because *P. promelas* males defend nests composed of floating or submerged substrate (Unger, 1983), a PVC breeding tile was placed in each of the distal tank compartments (McMillan & Smith, 1974). At the start of a trial, one size‐matched (<5 mm difference in SL) exposed male (MP_Virgin_, MP_EE2 10_, or MP_EE2 50_) and one control male were introduced to the distal compartments and given 3 h to acclimate to the test conditions (mean male standard length (mm) ± SD: MP_Virgin_ exposed vs. control: 52.09 ± 3.42 vs. 52.77 ± 3.27; MP_EE2 10_ exposed vs. control: 51.93 ± 3.64 vs. 52.77 ± 3.68; MP_EE2 50_ exposed vs. control: 52.23 ± 4.43 vs. 52.67 ± 4.46). A female (exposed or control) was then introduced to the middle compartment and given 1 h to acclimate before the trial began (mean female standard length (mm) ± SD: MP_Virgin_: exposed, 45.30 ± 8.89 and control, 42.67 ± 3.94; MP_EE2 10_: exposed, 41.24 ± 2.70 and control, 43.23 ± 3.38; MP_EE2 50_: exposed, 43.50 ± 2.89 and control, 44.79 ± 3.12). During the acclimation period, the trial participants were prevented from seeing or interacting with each other by the opaque barriers separating the compartments.

After the acclimation period, the opaque barriers were removed, and the female was permitted to interact with both males through the mesh barriers for 10 min. Trials were recorded using a GoPro Hero5 for later scoring of behavior. Each set of paired males was used twice: once with a control focal female and once with an exposed focal female from the same exposure trial series (e.g., MP_Virgin_, MP_EE2 10_, or MP_EE2 50_). The order of female presentations (control and exposed) and presence of males (control and exposed) in the left versus right compartment were randomized across all trials. Following the trial, fish were removed from the trial tank for morphological measurement.

### 2.4 Measurement of male sexually selected traits and behavioral variables

The intensity of melanic black body coloration of both males was scored by eye following Unger (1983) and modified as appropriate to score the head, midbody, and tail sections separately on a scale of 0 (no color) to 3 (maximally dark bars). Scoring was conducted immediately following a trial. The separate body coloration scores were added together to obtain a total coloration score for each male in each trial (maximum possible score: 9). Because males were used twice, a male's scores from both trials that he participated in were averaged prior to analysis. The number of tubercles was also counted for each male.

The following female behavioral response variables were scored from the tapes: (i) the total duration of time spent by a female with each male during the trial (defined as being within 7.5 cm of the male's compartment), and (ii) the number of female visits to each male. For each trial, the courtship behavior of each male was also quantified as the total duration of time each male spent interacting with the female while she was within 7.5 cm of his compartment. A male was considered to be interacting with the female if he was oriented toward the mesh barrier and performed courtship behaviors, such as approaching and/or attempting to contact the female. For each trial, overall female choice was determined based on side association data. In many species, females spend more time near males that they ultimately choose to spawn with (time with preferred male > time with non‐preferred male; Aspbury & Basolo, 2002; Lehtonen & Lindström, 2008). In addition, we estimated the overall reproductive motivation of each female by calculating the total amount of time spent interacting with both males during the trial, as well as the total number of visits to both males. Male reproductive motivation was calculated by adding the total amount of time spent interacting with the control and exposed females in consecutive trials. Trials were scored by one observer who was blinded to the treatment and exposure status of any of the focal fish. At the completion of trials, all focal fish were euthanized via an overdose of MS‐222 and measured for total length (mm).

### 2.5 Data analysis

A total of 288 fish underwent the exposure regime. Of these fish, 19 died during the 30‐day feeding period (control females = 6, control males = 4, MP_Virgin_ females = 1, MP_Virgin_ males = 1, MP_EE2 10_ males = 1, MP_EE2 50_ females = 4, and MP_EE2 50_ males = 2). An additional eight males were not used because their a priori size‐matched control or exposed male died before the trial. A total of 129 behavioral trials were completed. Of these, three trials were discarded prior to analysis. Two trials (one MP_EE2 10_ trial and one MP_EE2 50_ trial) were discarded because the size difference between the paired males was found to be >5 mm. One MP_EE2 10_ trial was discarded because the female became wedged in the mesh barrier during the trial. Final sample sizes retained for analysis were as follows: MP_Virgin_, *n* = 44; MP_EE2 10_, *n* = 43; and MP_EE2 50_, *n* = 40.

Paired *t*‐tests were used to compare the differences between paired control and exposed males in standard length (mm) and male secondary sexual traits (e.g., number of tubercles and total body coloration) in each trial series. Male and female morphological and behavioral data were assessed prior to analysis to confirm that the data satisfied parametric assumptions of normality (Shapiro–Wilk tests) and homogeneity of variance (Levenes tests). Body color scores recorded for each male were averaged across the two trials that he participated in prior to analysis. We used the general linear model (GLM) procedure in SPSS (IBM, version 28) to evaluate among‐treatment differences in male size, and the intensity of expression of other male sexually selected phenotypic characters (body color intensity and number of tubercles) in control and exposed males. For these analyses, treatment was specified as a fixed factor and the phenotypic traits of interest (e.g., color scores and number of tubercles) were specified as dependent variables.

In fish species that exhibit active female choice, male courtship is commonly a primary determinant of male reproductive success (Svensson et al., 2010). Therefore, we similarly used GLMs based upon the total amount of time that focal males spent courting females (i.e., time spent interacting with control female + time spent interacting with exposed female) to (i) confirm that the control males used in each trial series performed similar amounts of courtship, and (ii) evaluate the effect of MP exposure on overall reproductive motivation of males. For these models, courtship time was specified as the dependent variable and treatment was specified as the fixed factor. Additionally, for each trial series *t‐*tests were conducted to compare (i) the total amount of courtship performed by paired control and exposed males, and (ii) the amount of courtship directed toward exposed versus control females.

Among‐group differences in overall female reproductive motivation were tested separately for control and exposed females via analogous GLMs conducted on the total amount of time spent interacting with both males (control + exposed) and the total number of visits made by females to available males in each trial. For these analyses, treatment was specified as the fixed factor. Evidence for a significant female preference for exposed versus control males within each trial series was tested via paired *t*‐tests based on the total duration of time spent by a female with each male during a trial, and the number of visits made by females to exposed versus unexposed males. Two‐tailed binomial tests were used to test the null hypothesis that the overall proportions of exposed and control males preferred by females in each treatment series were equal.

Last, ANCOVAs were conducted separately for each treatment (MP_Virgin_, MP_EE2 10_, MP_EE2 50_) to investigate whether non‐exposed and exposed females differed in their responses to male phenotypic traits (size, color, courtship, tubercles). For these models, we calculated the strength of female preference for the preferred male, regardless of male exposure status, as (*time spent with preferred − time spent with non‐preferred)/*(*time spent with preferred + time spent with non‐preferred*) and used the resulting value as the dependent variable. Female exposure status (exposed or non‐exposed) was included as a fixed factor. Male trait differentials (*trait value of preferred male − trait value of non‐preferred male*) were calculated for each trial for body size, total color score, number of tubercles, and total amount of courtship performed during the trial, and were specified as covariates. All main effects and trait × female exposure status interactions were included in the models.

## 3 RESULTS

### 3.1 Effects of exposure on male sexual secondary traits

**FIGURE 2:**
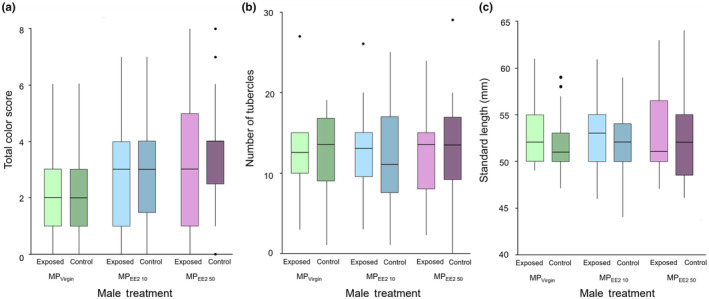
Boxplots comparing male phenotypic characteristics for (a) total body color score, (b) number of tubercles, and (c) standard length (mm). Males underwent 30‐day dietary exposure of microplastics (MPs) alone (MP_Virgin_) or with a low (MP_EE2 10_) or high (MP_EE2 50_) concentration of 17‐alpha ethinyl estradiol (EE2). Phenotypic characteristics were measured after behavioral trials ended on Day 31. Sample sizes are 20, 21, and 18 for the MP_Virgin_, MP_EE2 10_, and MP_EE2 50_ treatments, respectively. Boxes show the first quartile, median, and third quartile, and lines show the minimum and maximum data. Dots represent outliers.

The paired control and exposed males in each of the three exposure treatment series were similar in size (control vs. MP_Virgin_ exposed: *t* = −1.58, df = 21, *p* = .130; control vs. MP_EE2 10_ exposed: *t* = −0.542, df = 22, *p* = .593; control vs. MP_EE2 50_ exposed: *t* = −0.649, df = 19, *p* = .524). In addition, we found no evidence for differences between exposed and control males in any trial series in average body color score (control vs. MP_Virgin_ exposed: *t* = −0.026, df = 21, *p* = .795; control vs. MP_EE2 10_ exposed: *t* = 0.073, df = 22, *p* = .473; control vs. MP_EE2 50_ exposed: *t* = 0.411, df = 19, *p* = .686) or the extent of tubercle development (control vs. MP_Virgin_ exposed: *t* = −0.038, df = 21, *p* = .970; control vs. MP_EE2 10_ exposed: *t* = −0.365, df = 22, *p* = .719; control vs. MP_EE2 50_ exposed: *t* = 0.509, df = 19, *p* = .617; Figure 2).

General linear models conducted separately for control and exposed males revealed there were no differences among males in the MP_Virgin_, MP_EE2 10_, and MP_EE2 50_ trial series in either standard length or the number of tubercles (control males: standard length: *F*_2,62_ = 0.015, *p* = .985; tubercles: *F*_2,62_ = 0.170, *p* = .844; exposed males: standard length: *F*_2,62_ = 0.047, *p* = .954; tubercles: *F*_2,62_ = 0.075, *p* = .928). However, there was a significant difference among treatments in the expression intensity of body color for both control males (*F*_2,60_ = 6.065, *p* = .004) and exposed males (*F*_2,60_ = 3.637, *p* = .032). Post hoc tests (Tukey) indicated that both control (*p* = .003) and exposed (*p* = .026) males in the MP_EE2 50_ trial series were significantly darker than those in the MP_Virgin_ trial series (Figure 2).

### 3.2 Male behavior

**FIGURE 3:**
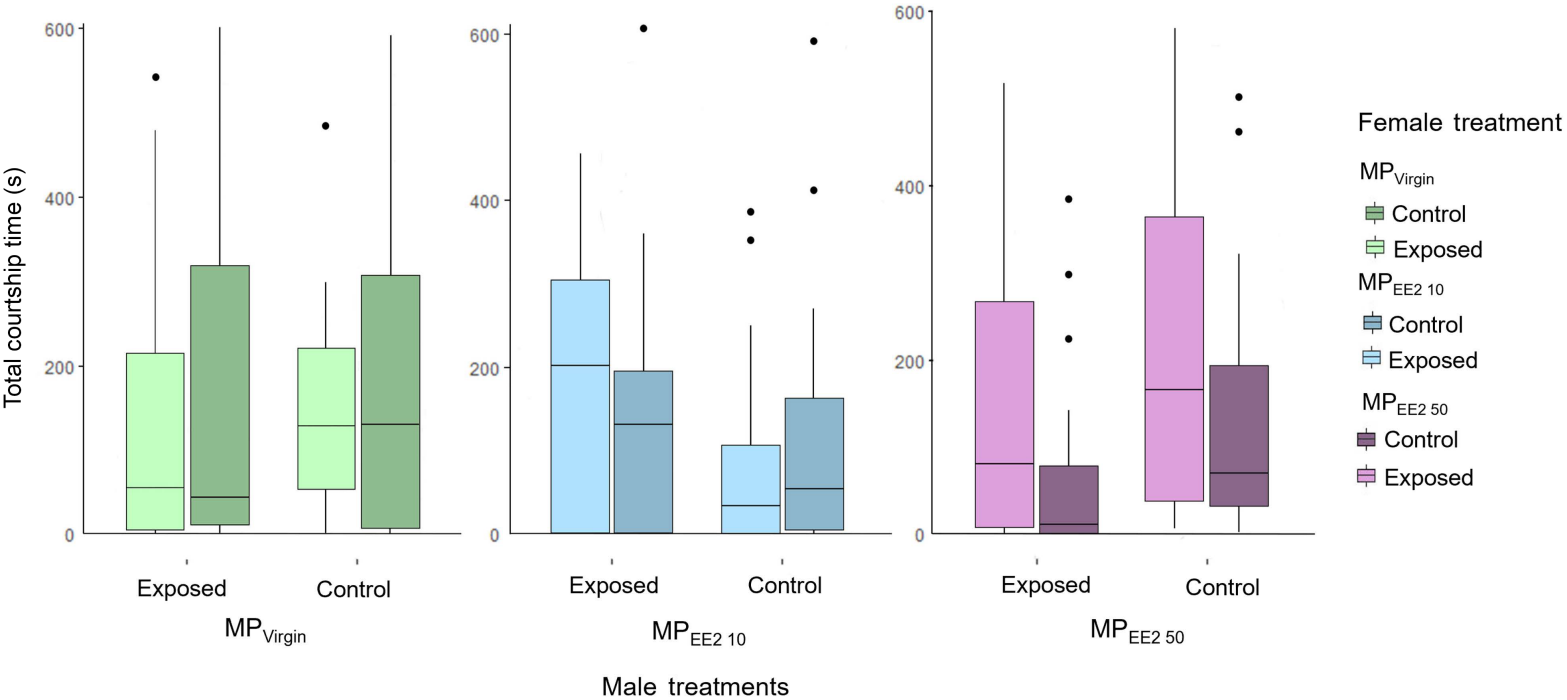
Boxplots showing the total duration of courtship performed by males toward control females, or females who underwent a 30‐day dietary exposure to microplastics (MPs) alone (MP_Virgin_) or MPs associated with a low (MP_EE2 10_) or high (MP_EE2 50_) concentration of 17‐alpha ethinyl estradiol (EE2). Sample sizes are 20, 21, and 18 for the MP_Virgin_, MP_EE2 10_, and MP_EE2 50_ treatments, respectively. Boxes show the first quartile, median, and third quartile, and lines show the minimum and maximum data. Dots represent outliers.

Among‐group comparisons of the total amount of time that males spent courting during trials revealed that the reproductive motivation of both control (*F*_2,123_ = 2.927, *p* = .057) and exposed males (*F*_2,123_ = 0.817, *p* = .444) was high and similar among trial series (Figure 3). Control males in the MP_Virgin_, MP_EE2 10_, and MP_EE2 50_ groups spent 25.8%, 16.4%, and 29.5% of overall trial time, respectively, engaged in interaction with females. Exposed males in the MP_Virgin_, MP_EE2 10_, and MP_EE2 50_ groups spent an average of 23.9%, 27.3%, and 19.7% of trial time engaged in courtship, respectively. A follow‐up analysis directly comparing the courtship activity of control and exposed males also indicated that males from all treatments exhibited a similar level of reproductive motivation (*F*_2,249_ = 0.866, *p* = .422). Comparisons of the behavior of control and exposed males within trials similarly indicated that exposure to MPs had little effect on a male's motivation to court compared to control males; the total duration of courtship performed by control versus exposed males toward both stimulus females (i.e., time spent courting control + exposed female) was similar in each treatment (MP_Virgin_: *t* = 0.500, df = 20, *p* = .622; MP_EE2 10_: *t* = −0.858, df = 19, *p* = .402; MP_EE2 50_: *t* = −1.914, df = 18, *p* = .072).

Within each trial series, *t*‐tests comparing the amount of time a male spent courting an exposed female versus a control female indicated that neither control nor exposed males in any treatment discriminated against exposed females (MP_Virgin_ trial series: time spent by control male courting exposed vs. control females: *t* = 1.219, df = 20, *p* = .237; by exposed males: *t* = −0.587, df = 20, *p* = .564; MP_EE2 10_ trial series: time spent by control male courting exposed vs. control females: *t* = −0.165, df = 20, *p* = .870; by exposed male: *t* = −1.434, df = 19, *p* = .168; MP_EE2 50_ trial series: time spent by control male courting exposed vs. control females: *t* = 0.534, df = 18, *p* = .600; by exposed male: *t* = −2.012, df = 18 *p* = .059; Figure 3).

### 3.3 Female behavior and mate choice

**FIGURE 4:**
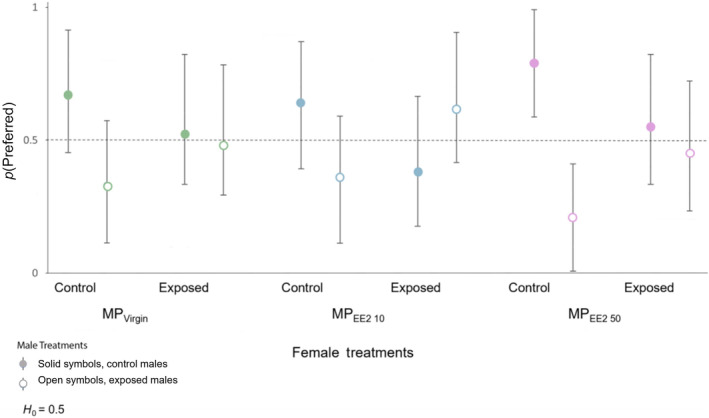
The relative proportions of control (solid markers) or exposed (open markers) males preferred by females in choice trials based on association time (time spent with preferred > time spent with non‐preferred). Exposed males and females underwent 30‐day dietary exposure of MPs alone (MP_Virgin_) or with a low (MP_EE2 10_) or high (MP_EE2 50_) concentration of 17‐alpha ethinyl estradiol (EE2). Error bars represent exact binomial 95% confidence intervals. Control groups: MP_Virgin_, *n* = 19; MP_EE2 10_, *n* = 20; MP_EE2 50_, *n* = 17 and exposed groups: MP_Virgin_, *n* = 21; MP_EE2 10_, *n* = 19; MP_EE2 50_, *n* = 18.

**FIGURE 5:**
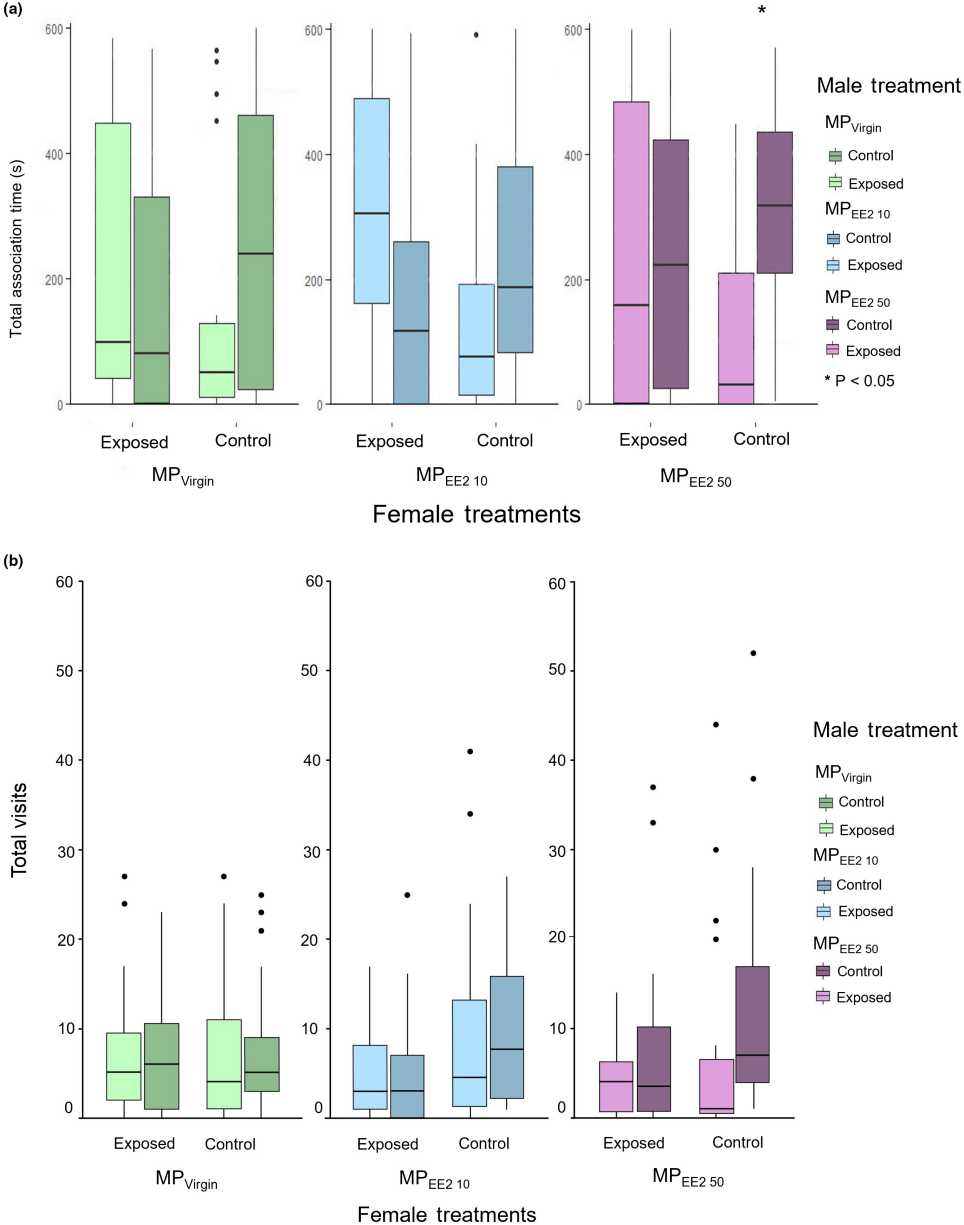
Boxplot showing (a) total duration of time spent and (b) total number of visits to paired exposed or control males by females in dual‐choice mate choice trials. Boxes show the first quartile, median, and third quartile, and lines show the minimum and maximum data. Dots represent outliers. MP_Virgin_, *n* = 21; MP_EE2 10_, *n* = 20; MP_EE2 50_, *n* = 19.

GLMs conducted separately for control and exposed females based on the total amount of time that females spent interacting with or visiting both males during trials revealed that both control and exposed females readily engaged in social interactions with males during trials. Control females in the MP_Virgin_, MP_EE2 10_, and MP_EE2 50_ treatments spent an average of 64.6%, 66.6%, and 66.6% of overall trial time, respectively, associating with males (*F*_5,120_ = 1.509, *p* = .192). Control females in each of the three trial series also made similar numbers of total visits to males (*F*_5,120_ = 1.757, *p* = .127). We did not detect a significant effect of MP treatment on either the number of visits (*F*_2,61_ = 1.039, *p* = .360) or total duration of time spent interacting with males (*F*_2,61_ = 1.896, *p* = .159) across the three trial series, with exposed females in the MP_Virgin_, MP_EE2 10_, and MP_EE2 50_ treatments spending an average of 68.5%, 75.8%, and 83.3% of overall trial time, respectively, associating with males. A follow‐up multivariate GLM based upon both the total number of male visits and total duration of time spent interacting with males also indicated that overall reproductive motivation was similar among control and exposed females (Wilk's λ = 0.918, *F*_6,242_ = 1.755, *p* = .109). Within trial series, exposed and control females differed in their responses toward exposed versus control males. Converting female association time into a binary metric of mate choice (i.e., time spent preferred > time spent non‐preferred) indicated that control females preferred control males in 67%, 64%, and 79% of trials in the MP_Virgin_, MP_EE2 10_, and MP_EE2 50_ treatments, respectively, although the null hypothesis that females would equally prefer control and exposed males was only rejected for the MP_EE2 50_ treatment (binomial tests: MP_Virgin_, *p* = 1.00; MP_EE2 10_, *p* = .824; MP_EE2 50_ = *p* = .019; Figure 4). By contrast, exposed females preferred control males in 52%, 38%, and 55% of trials in the MP_Virgin_, MP_EE2 10_, and MP_EE2 50_ treatments. For these focal females, the null hypothesis that control and exposed males would be selected with equal frequency was retained (binomial tests: MP_Virgin_, *p* = 1.00; MP_EE2 10_, *p* = .383; MP_EE2 50_ = *p* = .824; Figure 4). Further examination of the patterns in the amount of time spent by control and exposed females with control versus exposed males indicated that control females in all three treatments spent more time associating with control males than their size‐matched, exposed counterparts (Figure 5), although this trend only reached significance in the MP_EE2 50_ group (MP_Virgin_ trial series: *t* = 1.358, df = 20, *p* = .190; MP_EE2 10_: *t* = 1.309, df = 21, *p* = .205; MP_EE2 50_: *t* = 2.713, df = 18, *p* = .014). By contrast, patterns of mate association across trial series were inconsistent for exposed females, with females in the MP_Virgin_ and MP_EE2 10_ treatments spending a similar amount, or more, time with the exposed male compared to the control male (MP_Virgin_ trial series: *t* = −0.070, df = 22, *p* = .492; MP_EE2 10_: *t* = −1.770, df = 20, *p* = .092; MP_EE2 50_: *t* = 0.164, df = 19, *p* = .872; Figure 5a).

Similarly, there was no difference between the number of visits made by control females to control versus exposed males (MP_Virgin_ trial series: *t* = 0.213, df = 20, *p* = .833; MP_EE2 10_: *t* = 0.647, df = 21, *p* = .524; MP_EE2 50_: *t* = 1.448, df = 18, *p* = .165) or by exposed females to exposed vs. unexposed paired males (MP_Virgin_ trial series: *t* = −0.160, df = 22, *p* = .874; MP_EE2 10_: *t* = −0.079, df = 20, *p* = .938; MP_EE2 50_: *t* = 1.585, df = 19, *p* = .130; Figure 5b).

ANCOVAs conducted for each treatment, specifying female exposure status (exposed vs. non‐exposed) as a fixed factor and male phenotypic trait differentials as covariates did not find evidence that exposed and non‐exposed females differed in how they used the male traits measured to make choice decisions (Table 1).

**TABLE 1:** Results of ANCOVAs investigating differences in male assessment by exposed and non‐exposed females.

Factor	Treatment					
	MP_Virgin_		MP_EE2 10_		MP_EE2 50_	
	*F*	*p*	*F*	*p*	*F*	*p*
Female exposure status	0.018	.90	0.261	.61	0.033	.86
Male size	1.651	.21	0.074	.79	0.419	.52
Male color	1.334	.26	0.712	.41	0.266	.61
Male courtship	2.599	.12	0.156	.20	5.423	**.03**
Male tubercles	0.216	.65	0.054	.82	0.151	.70
Exposure status × size	0.284	.60	1.013	.32	0.362	.55
Exposure status × color	4.147	.05	0.614	.44	0.302	.59
Exposure status × courtship	0.486	.49	1.602	.21	0.373	.55
Exposure status × tubercles	0.000	.99	2.542	.12	0.865	.36

## 4 DISCUSSION

Recent evidence has shown the detrimental impacts of MPs on the behavior and performance of aquatic organisms in a variety of important contexts, including swimming, predator–prey interactions, and foraging (Carrasco et al., 2019; Ferreira et al., 2016; Suwaki et al., 2020; Wen et al., 2018; Yin et al., 2018), yet little is known about the effects of MPs on intraspecific social interactions in fish. Three findings emerged from this study that are relevant to this knowledge gap: first, somewhat surprisingly, male reproductive behavior and the expression of male secondary traits were not affected by exposure to MPs, alone or in combination with EE2. Second, despite this finding, non‐exposed females showed a preference trend for non‐exposed males, but exposed females did not. Third, the non‐exposed female preference for a non‐exposed male was most pronounced in the MP_EE2 50_ group, suggesting that MPs may serve as a vector for other contaminants known to alter social behavior and reproductive decision‐making. Taken together, these results suggest that exposure to MPs has the potential to alter intraspecific interactions in aquatic populations.

In our study, 30‐day dietary exposure to MPs had little effect on the expression of male sexually selected phenotypic traits, including courtship behavior. Similar results were recently reported by Swank et al. (2022), who found that exposure to MPs did not influence male aggression in *P. promelas*, or the ability of exposed males to compete for and acquire a territory. However, other studies have reported changes in other behaviors associated with MP exposure, including the suppression of shoaling in zebrafish (*Danio rerio*) and medaka (*Oryzias latipes*; Santos et al., 2022; Takai et al., 2022), increases in boldness in gilt‐head bream (*Sparus aurata*; Rios‐Fuster et al., 2021) and decreases in gastropod antipredator responses (Seuront, 2018). Moreover, in our study, exposure to EE2 via MP ingestion did not result in observable reductions in the expression of body color, number of tubercles, or courtship intensity, despite well‐known effects of estrogen exposure on these traits in teleost fishes (e.g., Gonzalez et al., 2021; Lavelle & Sorensen, 2011; Rahman et al., 2022; Salierno & Kane, 2009).

One potential explanation for the similarities in reproductive behavior observed among non‐exposed and exposed males could be the levels of circulating androgens in the males in our study. All the males used in trials were highly dominant individuals, which has been shown to correlate with high levels of testosterone and 11‐ketotestosterone (Parikh et al., 2006; Taves et al., 2009). It is possible that high levels of androgens in these males offset any effects of estrogen exposure. There is some evidence that individuals in population may vary in their degree of susceptibility to EDCs according to social status; for example, Ianova et al. (2017) found that dominant and subordinate males within a social hierarchy differ in how strongly they are affected by EDCs, with subordinate males (presumably with lower levels of circulating androgens) suffering more pronounced effects of exposure. Another potential explanation for these findings could be agonistic effects of MPs and EE2. Whereas some studies have found that co‐exposure with MPs enhanced the effects of other contaminants (Gu et al., 2020; Wang et al., 2022), others have reported the opposite effect (Elizalde‐Velázquez et al., 2022; Kaur et al., 2022). For example, Oliveira et al. (2013) found a reduction in pyrene‐related mortality in the presence of pyrene and MPs in the common goby.

Even though we did not find observable effects of exposure on male traits used by females in making mate choice decisions, non‐exposed females in all three treatments consistently spent more time associating with non‐exposed males compared with exposed males. The magnitude of this difference was strongest in the MP_EE2 50_ group, with control males being preferred significantly more often than their exposed counterparts. These results are in accordance with the results of previous studies in fish and frogs showing that females frequently discriminate against males exposed to estrogens and other EDCs (Dzieweczynski & Kane, 2017; Hoffmann & Kloas, 2012; Partridge et al., 2010). For example, Baatrup and Henriksen (2015) found that the reproductive behavior of unexposed female zebrafish was significantly decreased when paired with EE2‐exposed males. Given that we did not observe exposure‐induced changes in the expression of male secondary sexual traits known to impact female decision‐making (Bakker & Mundwiler, 1994; Jacob et al., 2009; Takashi, 2018), or reductions in courtship intensity (Colman et al., 2009), the phenotypic assessment traits underpinning this result are unclear. One possibility is that females cued in on an aspect of courtship behavior not considered in the scope of this study, such as quality of performance rather than intensity. In many fish species, the strength of female preference for a given male is accurately predicted by differences among males in the overall amount of courtship demonstrated (Hermann et al., 2015; Ward & McLennan, 2009; Ward & Blum, 2012; but see Lehtonen, 2012 for an example of a species in which this is not the case). However, the courtship repertoire of male fathead minnows includes several behavioral elements, including leads to the mating tile, physical contact with the female, and chases (Weinberger II & Klaper, 2014). Thus, although males in all treatments performed similar durations of courtship, females may have also attended to differences among males in courtship performance, rather than overall duration (Baldauf et al., 2013).

By contrast, females who were exposed to MPs were more likely to approach and associate with both stimulus males. Because the phenotypic traits used by females to evaluate male quality are finely attuned to local environmental conditions, female mate choice is easily influenced by anthropogenic change (Tomkins et al., 2016; Wong & Candolin, 2015). Our results are consistent with previous studies that have found that exposure to EDCs alters the discriminatory capacity of females. For example, short‐term exposure to bisphenol A was shown to lead to reduced discrimination and weakened reproductive isolation between closely related *Cyprinella* species (Ward & Blum, 2012). Similar results were observed in female guppies exposed to androgenic 17 beta‐trenbolone (TB); exposed females were not as selective when choosing between exposed and unexposed males (Tomkins et al., 2016). Taken together, this growing evidence suggests that additional research is warranted to better understand EDC‐induced changes in neural and endocrine mechanisms that might contribute to relaxed female assessment and cognitive changes in decision‐making.

Other asymmetric sex‐specific responses have been observed after exposure to EDCs or MPs. Sex‐specific effects after exposure to TB and EE2 have been reported, with female guppies (*Poecilia reticulata*) demonstrating increases in risk‐taking behavior after exposed to EE2 or TB, whereas exposed males showed decreases in the same behaviors (Heintz et al., 2015). Female Eastern mosquitofish (*Gambusia holbrooki*) exposed to TB also showed a decrease in the amount of time that they interacted with males compared to non‐exposed fish, whereas exposed and non‐exposed males showed no differences in reproductive behavior (Saaristo et al., 2013). Although there is limited research evaluating the potential for sex‐specific effects of MP exposure on aquatic species, male *Drosophila melanogaster* were reported to have a higher mortality than female flies after exposure (Kholy & Naggar, 2023). In the common European hermit crab, *Pagurus bernhardus*, exposure to MPs decreased the time taken by females to select and enter shells compared to males; however, they were also more likely to choose high‐quality shells (McDaid et al., 2023).

Anthropogenic activities may influence aquatic ecosystems by altering functional roles within communities (Lomartire et al., 2021). Fathead minnows are a common forage fish species, population declines of which can adversely impact species at higher trophic levels (Kidd et al., 2007). Thus, the findings of this study may have implications for understanding and mitigating risks to populations affected by anthropogenic contaminants, such as MPs and EDCs, and may help to maintain the structure of communities in natural environments. To better understand the short‐ and long‐term ecological and evolutionary effects of MPs and co‐occurring contaminants on wildlife, additional research is needed (Wang, Li, et al., 2019). Future research should prioritize studies aimed at uncovering the mechanisms underlying changes in intraspecific behavior and the outcomes of intraspecific interactions (Swank et al., 2022). In addition, research should be undertaken to understand whether the effects of exposure on reproductively motivated males and females are retained, or even enhanced, in offspring (Zhou et al., 2020).

## AUTHOR CONTRIBUTIONS

**Grace Carter:** Conceptualization (equal); data curation (equal); formal analysis (lead); methodology (equal); visualization (equal); writing – original draft (lead); writing – review and editing (equal). **Jessica Ward:** Conceptualization (equal); data curation (equal); formal analysis (supporting); funding acquisition (lead); methodology (equal); project administration (lead); supervision (lead); writing – original draft (supporting); writing – review and editing (equal).

## CONFLICT OF INTEREST STATEMENT

The authors have no competing interests relating to the work submitted for publication.

## Data Availability

Data are available through Dryad (https://doi.org/10.5061/dryad.j3tx95xmd).
